# Effectiveness of Unguided Internet-Based Cognitive Behavioral Therapy and the Three Good Things Exercise for Insomnia: 3-Arm Randomized Controlled Trial

**DOI:** 10.2196/28747

**Published:** 2022-02-09

**Authors:** Daisuke Sato, Yoichi Sekizawa, Chihiro Sutoh, Yoshiyuki Hirano, Sho Okawa, Motohisa Hirose, Ryo Takemura, Eiji Shimizu

**Affiliations:** 1 Department of Cognitive Behavioral Physiology, Graduate School of Medicine Chiba University Chiba Japan; 2 Cognitive Behavioral Therapy Center Chiba University Hospital Chiba Japan; 3 Research Institute of Economy Trade and Industry Tokyo Japan; 4 Research Center for Child Mental Development Chiba University Chiba Japan; 5 United Graduate School of Child Development Osaka University, Kanazawa University, Hamamatsu University School of Medicine, Chiba University and University of Fukui Suita Japan; 6 Department of Preventive Medicine and Public Health Keio University School of Medicine Tokyo Japan

**Keywords:** insomnia, internet-based treatment, cognitive behavioral therapy, positive psychology, randomized controlled trial, mobile phone

## Abstract

**Background:**

The treatment of insomnia with sleep medication causes problems such as long-term use, dependence, and significant economic losses, including medical expenses. Evidence-based lifestyle guidance is required to improve insomnia symptoms not only in person but also in easy-to-use web-based formats.

**Objective:**

This study aims to clarify whether unguided internet-based cognitive behavioral therapy (ICBT) or the Three Good Things (TGT) exercise, both administered as self-help internet interventions without email support, could improve insomnia symptoms compared with a waiting list control (WLC) group.

**Methods:**

A 4-week program was implemented, and participants were randomly allocated to 1 of the 3 groups. The primary outcome measure was the Pittsburgh Sleep Questionnaire (PSQI) score at 4 weeks compared with baseline.

**Results:**

Of the 21,394 individuals invited to participate, 312 (1.46%) met the eligibility criteria and were randomly assigned to 1 of the 3 groups. Of these 312 individuals, 270 (86.5%; ICBT 79/270, 29.3%; TGT 88/270, 32.6%; and WLC 103/270, 38.1%) completed a postintervention survey at 4 and 8 weeks. The adjusted mean changes of the primary outcome measure (PSQI) in the ICBT (−1.56, 95% CI −2.52 to −0.59; *P*<.001) and TGT (−1.15, 95% CI −2.08 to −0.23; *P*=.002) groups at 4 weeks from baseline showed a significant improvement compared with the WLC group. The adjusted mean changes in the secondary outcome measures of sleep onset latency, total sleep time, Athens Insomnia Scale score, and Patient Health Questionnaire-9 score at 4 weeks from baseline, as well as in the PSQI at 8 weeks from baseline, showed significant improvement for ICBT. Moreover, total sleep time, Athens Insomnia Scale, and Patient Health Questionnaire-9 scores at 4 weeks from baseline showed a significant improvement in the TGT group compared with the WLC group.

**Conclusions:**

A total of 4 weeks of unguided ICBT and TGT exercises improved insomnia.

**Trial Registration:**

University Hospital Medical Information Network Clinical Trial Registry UMIN000034927; https://center6.umin.ac.jp/cgi-open-bin/ctr_e/ctr_view.cgi?recptno=R000039814

## Introduction

### Background

Insomnia describes the inability to sleep, including difficulty falling asleep, difficulty staying asleep, difficulty sleeping, and early waking, despite an attempt to sleep at the right time and in the right environment. This condition reduces an individual’s quality of life by causing various daytime dysfunctions, including sleepiness during the day [1]. Insomnia can be caused by a variety of factors, including changes in social life such as decreased interpersonal interaction and socializing, as well as mental and physical illnesses, decreased physical function, and physiological changes in the brain. Insomnia impairs daytime activity and triggers additional illnesses such as depression, lifestyle-related diseases, and cancer. Insomnia is a common complaint in adults, with 13.5% to 20.6% of the general population experiencing daytime sleepiness >3 times per week [2]. It has been reported that 14.9% of people in Japan have profound daytime sleepiness [3]. Another study found that 1 in 5 Japanese adults had sleep problems [4]. In Japan, the economic losses caused by sleep deprivation and insomnia have been estimated to be 15 trillion yen (US $8.67 billion) [5,6].

From the perspective of optimizing medical costs associated with lifestyle-related diseases, such as diabetes, hypertension, and hyperlipidemia, lifestyle guidance is increasingly being introduced as a complement to the use of medication. However, the evidence base to support the introduction of lifestyle guidance is lacking. The provision of face-to-face advice paired with an insomnia improvement program delivered as easy-to-use internet-based life guidance will likely reduce medical expenses.

Insomnia practice guidelines in the United States and Australia recommend cognitive behavioral therapy (CBT) as the first choice for the treatment of insomnia. In Japan, there are very few medical institutions that can provide CBT, and there are no barriers to prescribing psychotropic drugs outside of psychiatry and psychosomatic medicine. However, pharmacotherapy is associated with side effects such as dependence, tolerance, anterograde amnesia, muscle relaxant effects, carryover effects, and rebound insomnia. Furthermore, it increases the risk of double prescription and overdose and requires long-term treatment. However, the success of pharmacotherapy is limited. According to the Japanese Society of Sleep Studies guidelines, it is preferable to prioritize CBT over pharmacotherapy for insomnia.

CBT for insomnia is a treatment that focuses on anxiety and biased thinking about sleep and improves insomnia by reviewing an individual’s lifestyle, existing anxiety and tension, and any thinking related to the maintenance of insomnia. Sleep hygiene instructions can provide important knowledge about sleep, including how to adjust living conditions so that good-quality sleep can be obtained, and practice measures for maintaining good-quality sleep throughout life. This study used internet-based CBT (ICBT) without email support to provide sleep hygiene guidance as a trial treatment. ICBT was paired with a self-help intervention as a form of noninvasive self-medication.

Positive psychology focuses on positive psychological traits such as happiness, optimism, and satisfaction with life and was developed by Seligman et al [7] of the University of Pennsylvania based on the reflection that traditional psychology was excessively focused on normalizing negative traits such as mental illness. Positive psychology is based on the idea of nurturing [7]. A typical method of positive psychology comprises the exercise of writing 3 good things every day before going to bed every night [7]. It is a simple diary-like exercise that involves listing 3 good things that happened that day and providing a written explanation of why those things happened. In this study, this exercise was called the Three Good Things (TGT) exercise.

ICBT has gained increasing attention as a possible treatment for improving insomnia, and many studies on ICBT have been conducted. ICBT provides web-based CBT and sleep hygiene guidance, simulating an ordinary CBT session (counseling) that is typically conducted by the patient and therapist face-to-face. With the approval of the Chiba University School of Medicine Hospital clinical trials ethics review board (approval number G27040), the combination of guided ICBT for insomnia and routine care (ie, usual care [UC]) has been shown to significantly reduce the Pittsburgh Sleep Quality Index (PSQI) score [8,9]. In a randomized controlled trial (RCT) of 23 patients with insomnia whose symptoms persisted even after taking sleep medications such as benzodiazepines, the adjusted mean change in total PSQI score from baseline to 6 weeks was −6.11 in the ICBT+UC group (n=11), which was significantly better than the 0.40 change seen in those who underwent UC alone (n=12; *P*<.001). In addition, a significant improvement was seen for the adjusted mean changes in PSQI score; sleep onset latency (SOL); sleep efficiency (SE); number of awakenings; and mean change in depression at 3, 6, and 12 weeks from baseline in the ICBT+UC group. No adverse events were reported.

Seligman et al [7] previously evaluated the effects of the TGT exercise in a RCT. According to that study, the group that performed this exercise daily for 1 week not only increased self-reported levels of happiness but also reported decreased depression compared with the control group, and the effect persisted after 6 months. To the best of our knowledge, no studies have examined whether the TGT exercise can reduce insomnia. However, recent reviews have shown that there is a correlation between positive emotions and sleep [10]. TGT may improve sleep by improving positive emotions.

### Objectives

This study aims to determine the effectiveness of an unguided, web-based self-help intervention in reducing insomnia. Using an RCT design, a nonclinical population of adults with insomnia was allocated to one of three groups: (1) the ICBT group, (2) the TGT group, and (3) the waitlist group. It is hypothesized that unguided ICBT and TGT would result in higher quality sleep in the treatment groups than in the waitlist group.

## Methods

### Trial Design

We report this RCT trial in accordance with the CONSORT-EHEALTH (Consolidated Standards of Reporting Trials of Electronic and Mobile Health Applications and Online Telehealth) version 1.6 checklist (Multimedia Appendix 1).

This study comprised an exploratory, parallel-group (3 groups), randomized, open-label, controlled study, incorporating a nonintervention group (waiting list control [WLC]). The registration of the participants was started in February 2019.

### Participants and Recruitment

An email was sent to the registered monitors owned by the internet research company that commissioned the research, and a preliminary survey was conducted on the internet in relation to the inclusion and exclusion criteria given in the following sections, after which informed consent was obtained.

Potential participants were required to meet all the following inclusion criteria at the time of the preliminary survey: (1) aged 20 to 70 years (regardless of sex); (2) patients with mild or severe sleep disorders, who scored ≥6 on the Athens Insomnia Scale (AIS) and ≥6 on the PSQI; (3) sleep disorder occurring at least three nights per week and lasting for at least 3 months; (4) access to internet use with PCs, smartphones, and tablets; and (5) understood the explanations in Japanese and freely provided web-based consent.

Exclusion criteria of the study included (1) those with moderate or severe anxiety symptoms, scoring ≥10 on the Generalized Anxiety Disorder-7 (GAD-7) questionnaire; (2) those with moderate or severe depression symptoms, scoring ≥10 points on the Patient Health Questionnaire-9 (PHQ-9); (3) the confirmed presence of alcohol or drug dependence, diagnosed by a medical institution within the past year, excluding tobacco smokers; (4) suicidal thoughts, with a score of ≥2 in the Q9 part of the PHQ-9; (5) the confirmed diagnosis of cerebral organic diseases such as sleep apnea syndrome, restless leg syndrome, epilepsy, dementia, or cerebrovascular disease; (6) the confirmed diagnosis of a severe progressive physical illness, such as cancer or heart failure; (7) the use of pharmacotherapy for mental disorders, including insomnia symptoms; and (8) night shift work between 8 PM and 2 AM.

Individuals who met the above conditions and provided consent were enrolled as study participants. This study excluded patients commonly treated with pharmacotherapy for insomnia.

### Case Registration and Allocation Methods

Case registration was performed via the trial website. Among the registered members (candidate participants) of monitors owned by the internet research company, those who met the conditions were automatically assigned participant identification codes when they accessed the servers. Members voluntarily underwent a preliminary survey after explanation and agreement, following the distribution of the recruitment materials. In the first preliminary survey, AIS, PSQI, GAD-7, and PHQ-9 were administered to select and screen participants for inclusion. Only those who met the inclusion criteria and did not meet any of the exclusion criteria were able to proceed to the next preliminary survey. The second preliminary survey was conducted 2 weeks after the first preliminary survey and contained the same content as the first survey. Only those who met the inclusion criteria and did not meet any of the exclusion criteria proceeded to take part in the study. Responses from the second preliminary survey were used in the analysis as baseline values before the intervention. After the participants were randomly assigned, the method of accessing the intervention program assigned to each participant was provided by email.

### Randomization

Allocation modifiers included baseline PSQI scores (≥12 and ≤11) and gender (male and female).

### Intervention

#### Intervention Schedule and Methods

Participants were randomly assigned to 1 of the 3 groups. The study period was 4 weeks; thus, the ICBT group underwent an unguided ICBT program for 4 weeks, the TGT group underwent a TGT exercise program for 4 weeks, and the nonintervention group (WLC) waited for 4 weeks without an intervention. They then received access to their assigned unguided ICBT program and the TGT exercise program for 4 weeks, 7 times a week for 15 to 20 minutes each time.

The intervention programs comprised the following two types: unguided ICBT program and TGT program.

#### Unguided ICBT Program

We encouraged participants to take part in the unguided ICBT program for 4 weeks. Participants accessed the ICBT site and performed the following tasks autonomously:

1. Week 1 session: keeping a sleep diary (understanding the significance of the sleep diary and how to write it)
2. Week 2 session: changing behavior (improving behavioral habits that maintain insomnia, using stimulus control)
3. Week 3 session: capturing thoughts (reconstructing cognition related to sleep using the column method)
4. Week 4 session: changing sleep time (adjusting sleep schedule using the sleep restriction method)

#### TGT Program

Participants were encouraged to participate in the TGT exercise for 4 weeks. The participants who accessed the site received the prompt by email and autonomously filled out the 3 good things on that day. An explanation of the methods of the TGT exercise is provided elsewhere by Seligman et al [7] and Seligman [11].

### Measures

#### Evaluation Items

The primary outcome was the change in PSQI scores from baseline to the postintervention survey at 4 weeks. The secondary outcome was the change in PSQI scores from baseline to the postintervention survey at 8 weeks and changes in SOL, total sleep time (TST), SE, AIS, GAD-7, PHQ-9, and Center for Epidemiologic Studies–Depression Scale (CES-D) positive items only from baseline to the first and second postintervention surveys at 4 and 8 weeks. Web-based assessments were also administered.

#### PSQI Evaluation

The PSQI is a self-administered questionnaire for evaluating sleep and sleep quality [12]. Eighteen questions evaluated seven factors: sleep quality, sleep onset time, sleep time, SE, difficulty sleeping, use of sleep medication, and drowsiness during the day that hinders daily life. A total of 7 factors (0-3 points) were summed to calculate the total score (0-21 points). The higher the score, the greater the sleep impairment. A Japanese version has also been developed, with the cutoff set at 6 points [13].

#### AIS Evaluation

The AIS is a self-administered questionnaire for assessing the severity of insomnia [14]. It comprises eight items: sleep, midnight awakening, early morning awakening, TST, overall sleep quality, daytime satisfaction, physical and mental daytime activity, and daytime sleepiness. A total of 8 items (0-3 points) were summed to calculate the total score (0-24 points). A Japanese version has also been developed, with the cutoff set at 6 points [15].

#### GAD-7 Evaluation

The GAD-7 is a self-administered questionnaire for evaluating generalized anxiety disorders [16]. It comprises 7 items, and covers the 2 weeks immediately before the administration of the test. The scoring system is as follows: *No at all: 0 points*, *Several days: 1 point*, *More than half: 2 points*, and *Almost every day: 3 points*. The total score is calculated from 0 to 21 points. A Japanese version has also been developed, with 0 to 4 points suggesting no generalized anxiety disorder, 5 to 9 points representing mild generalized anxiety disorder, 10 to 14 representing moderate generalized anxiety disorder, and 15 to 21 indicating severe generalized anxiety disorder [17].

#### PHQ-9 Evaluation

The PHQ-9 is a self-administered questionnaire for evaluating major depressive disorder [18]. It comprises 9 items and covers 2 weeks immediately before administration of the test. The scoring system is as follows: *No at all: 0 points*, *Several days: 1 point*, *Half or more: 2 points*, and *Almost every day: 3 points*. The total score is calculated from 0 to 27 points. A Japanese version has also been developed, with 0 to 4 points suggesting that the individual is not depressed, 5 to 9 points suggesting mild depression, 10 to 14 points suggesting moderate depression, 15 to 19 points suggesting moderate to severe depression, and 20 to 27 points suggesting severe depression [19].

#### CES-D Evaluation

The CES-D is a self-administered questionnaire for assessing depression [20]. It comprises 20 items about mood and physical condition covering the past week using a 4-point scale ranging from 0 (not at all) to 3 (over 5 days). In this study, 16 negative items were not used, and only positive items were used. The positive items comprised four statements: “I think I have the same ability as other people,” “I can think positively about the future,” “I can enjoy my life without complaints,” and “I enjoy every day.” The total score is calculated from 0 to 12 points. A Japanese version has also been developed, with a higher total score indicating a higher degree of positive emotions [21].

### Statistical Analysis

#### Overview

All participants who enrolled in this study, responded to the intervention program at least once after randomization, and had efficacy data were the most significant population for full analysis set (FAS). However, participants for whom baseline data could not be obtained and those who violated the dominant protocol (eg, violating the inclusion criteria or exclusion criteria and incorrect assignment to the intervention program) were excluded.

Participants selected from the FAS and those meeting the following criteria were selected as the target population per-protocol set (PPS) that complied with the study protocol: (1) completed at least 75% of the intervention program, (2) had available measurements of critical variables, and (3) had no major test plan violation such as selection criteria violation, exclusion criteria violation, or incorrect assignment to an intervention program.

The sample size was based on a previous study by van Straten et al [22], which indicated that the estimated difference in changes of PSQI scores from baseline was approximately 3.5. Assuming a group difference of 3.5 (SD 10.0) points, 66 participants per group provided 80% power to detect a difference in PSQI scores among the WLC, ICBT, and TGT groups at a 5% significance level.

#### Analysis of Participant Background

The distribution of participant background data and summary statistics for each analysis population were calculated for each group. For nominal variables, the frequency and proportion of categories have been shown for each group. For continuous variables, summary statistics (number of cases, mean, SD, minimum, median, and maximum) were calculated for each group. Pearson chi-square test was used for nominal variables, except when ≥20% of the cells had an expected frequency <5 (when Fisher exact test was used). 2-tailed Student *t* test, or Wilcoxon rank-sum test, were also used. The significance level was set at 5% for both sides.

#### Analysis of Effectiveness

The primary outcome of the efficacy of the interventions was the PSQI score, an index for improving insomnia. The primary purpose of this study was to examine the superiority of the ICBT and TGT groups in improving the insomnia status of nonclinical cases of insomnia compared with the WLC group. We estimated the difference in PSQI score change between the test and control groups and the 95% 2-sided CI. To test the null hypothesis that the changes in PSQI scores in both groups were equal in the primary analysis, covariance analysis was performed using the assignment adjustment factor as a covariate. The allocation adjustment factors were the PSQI score at the time of registration and gender. The significance level of the test was set at 5% (2-sided). Adjustment of multiplicity was performed using the Dunnett method, as a pairwise comparison of the 2 groups was performed for the control group.

The secondary evaluation items of effectiveness were analyzed to supplement the primary analysis results. No adjustment for multiplicity was made in the analysis of the secondary efficacy outcomes. The significance level of the hypothesis test was set at 5% (2-sided), and the two-sided 95% CI was calculated.

### Statement of Ethics

This study was approved by the clinical trials ethics review board of the Chiba University Hospital (registration number G30022) and was registered as a clinical trial (UMIN000034927). A document explaining consent was presented to the participants on a webpage, accompanied by a verbal explanation as part of a video animation by the principal investigator. After viewing these materials, individuals who freely agreed to participate were recruited into the study.

The authors assert that all procedures contributing to this work comply with the ethical standards of the relevant national and institutional committees on human experimentation and with the Helsinki Declaration of 1975, as revised in 2008.

## Results

### Trial Flow

**Figure 1:**
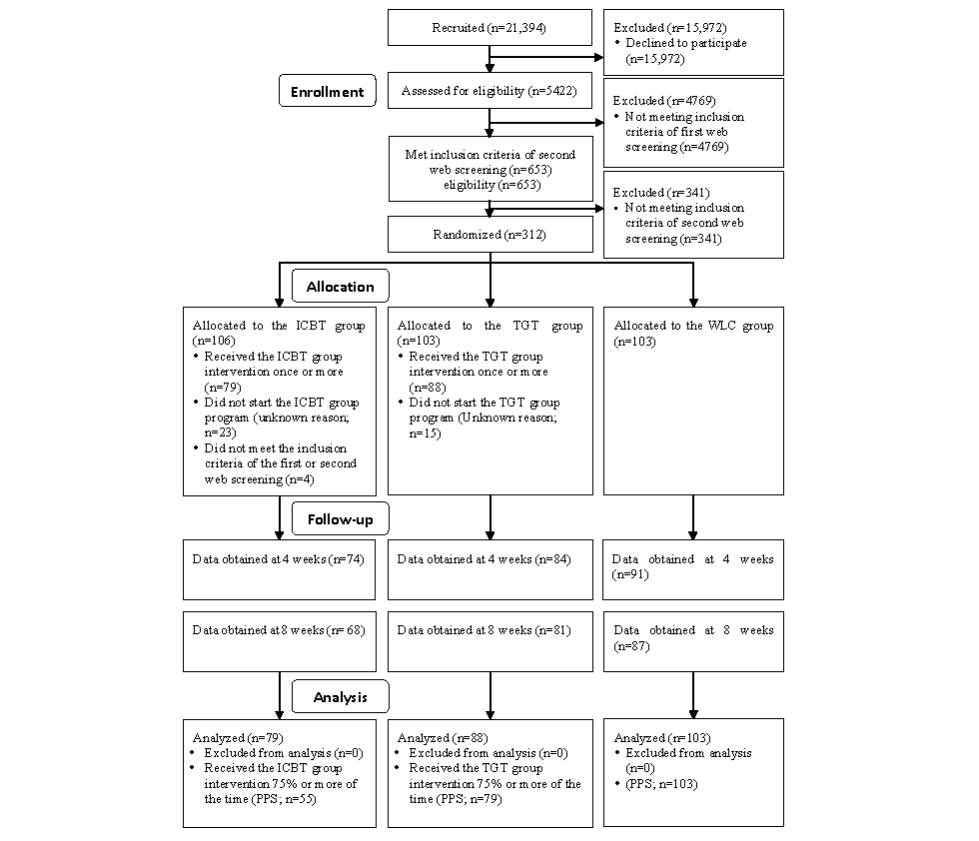
CONSORT (Consolidated Standards of Reporting Trials) diagram of participant flow throughout the study. ICBT: internet-based cognitive behavioral therapy; PPS: per-protocol set; TGT: Three Good Things; WLC: waiting list control.

Figure 1 shows the research flow based on the CONSORT (Consolidated Standards of Reporting Trials) guidelines. Of the 21,394 individuals contacted through the internet research company, consent to participate in the study was obtained from 312 (1.46%) individuals who met the eligibility criteria and were randomly assigned to the ICBT, TGT, or WLC group. Of the 106 people assigned to ICBT, 23 (21.7%) did not start the ICBT program at least once, and 4 (3.8%) who performed the ICBT program at least once were classified as trial deviations, leaving 79 (74.5%) individuals with a FAS.

For details of the deviation related to PSQI, there was an error in the formula set by the internet survey company that conducted the web questionnaire, and the correct score was ≤5; however, it was judged to be ≥6 by incorrect calculation. Therefore, of the 312 individuals, 4 (1.3%) individuals who had initially been selected as participants and who should not have been selected underwent the ICBT program; these individuals were reported as deviations to the Chiba University Hospital clinical trials ethics review committee and accepted as such.

Separately, there was an error in the formula used by the internet survey company that conducted the web questionnaire. In the error, the correct PSQI total score was ≥6; however, it was judged incorrectly to be ≤5. The correct PHQ-9 total score should have been calculated from the 1st to 9th items (the 10th item should have been excluded); however, the internet survey company that conducted the web questionnaire mistakenly calculated from the 1st to the 10th item. In addition, the correct GAD-7 total score should have been calculated from the first to seventh items (the eighth item was not added); however, the internet survey company that conducted the web questionnaire mistakenly calculated from the first to eighth items. For these reasons, of the 21,394 individuals, 83 (0.39%) were not selected in the first screening, and 25 (0.12%) were not selected in the second screening.

Of the 106 participants assigned to the ICBT group, 74 (69.8%) provided data at 4 weeks, 68 (64.2%) provided data at 8 weeks, and 55 (51.9%) had an attendance of ≥75% during the ICBT program; thus, the PPS was 55. Of the 103 participants assigned to the TGT group, 15 (14.6%) were excluded who did not start the TGT program at least once; thus, the FAS was 88. In the TGT group, of the 103 participants, 84 (79.2%) provided data at 4 weeks, and 81 (78.6%) provided data at 8 weeks. Approximately 76.7% (79/103) of participants had an attendance of ≥75% during the TGT program; thus, the PPS was 79.

Of the 312 participants, 103 (33%) were assigned to the WLC group; thus, the FAS score for this group was 103. Data were obtained successfully for 88.3% (91/103) of these participants at 4 weeks and for 84.5% (87/103) of participants at 8 weeks; thus, the PPS was 103. The registration of participants started in February 2019, and the follow-up ended in May 2019.

There were no adverse events. When adverse events occurred for participants, we asked them to report the adverse events by email. However, there were no reports.

### Participant Characteristics

Table 1 shows the characteristics of the participants at baseline. Female participants comprised 41.5% (112/270; ICBT group 32/79, 41%; TGT group 39/88, 44%; and WLC group 41/103, 39.8%) of the study population, with a mean age of 50.4 (SD 10.8; ICBT group: 49.8, SD 11.1; TGT group: 50.5, SD 11.0; and WLC group: 51.0, SD 10.4) years. There were no significant differences in sex, age, marital status, educational history, or employment status among the 3 groups.

Table 2 presents the baseline data for the primary and secondary outcome measures. There were no significant differences among the 3 groups for the PSQI, AIS, GAD-7, or PHQ-9 scores.

**Table 1:** Participant characteristics (N=270).

Characteristics			ICBT^a^ group (n=79)		TGT^b^ group (n=88)		WLC^c^ group (n=103)		*P* value	
**Sex** **,** **n (%)**										.81
	Female	32 (40.5)		39 (44.3)		41 (39.8)				
	Male	47 (59.5)		49 (55.7)		62 (60.2)				
Age (years), mean (SD)			49.8 (11.1)		50.5 (11.0)		51.0 (10.4)		.78	
**Marriage, n (%)**										.26
	Single	19 (24.1)		18 (20.5)		25 (40.3)				
	Married	58 (73.4)		60 (68.2)		72 (37.9)				
	Divorce	2 (2.5)		10 (11.4)		6 (33.3)				
Education, mean (SD)			15.4 (1.9)		15.0 (2.0)		15.4 (2.1)		.33	
**Working, n (%)**										.71
	Full time	52 (65.8)		51 (57.9)		66 (39.1)				
	Part-time	11 (13.9)		14 (15.9)		18 (41.9)				
	Unemployed	16 (20.3)		23 (26.1)		19 (32.8)				

^a^ICBT: internet-based cognitive behavioral therapy.

^b^TGT: Three Good Things.

^c^WLC: waiting list control.

**Table 2:** Baseline data of primary outcome and secondary outcome (N=270).

Outcomes			Value, mean (SD)							*P* value
			ICBT^a^ group (n=79)		TGT^b^ group (n=88)		WLC^c^ group (n=103)			
Primary outcome: insomnia symptoms (PSQI^d^)			9.8 (2.4)		9.8 (2.0)		9.8 (2.3)		.97	
**Secondary outcome** **:** **sleep**										
	AIS^e^	10.2 (2.9)		10.0 (2.7)		10.2 (2.9)		.77		
	SOL^f^ (minutes)	41.6 (27.9)		47.8 (40.6)		41.2 (35.0)		.66		
	TST^g^ (hours)	318.6 (63.7)		323.8 (52.1)		319.9 (61.7)		.17		
	Sleep efficiency (%)	82.1 (15.2)		85.3 (10.9)		84.3 (14.7)		.55		
**Secondary outcome** **:** **mood**										
	GAD-7^h^	3.0 (2.5)		3.1 (2.3)		3.2 (2.5)		.92		
	PHQ-9^i^	4.5 (2.2)		4.6 (2.2)		4.4 (2.2)		.74		

^a^ICBT: internet-based cognitive behavioral therapy.

^b^TGT: Three Good Things.

^c^WLC: waiting list control.

^d^PSQI: Pittsburgh Sleep Quality Index.

^e^AIS: Athens Insomnia Scale.

^f^SOL: sleep onset latency.

^g^TST: total sleep time.

^h^GAD-7: Generalized Anxiety Disorder-7.

^i^PHQ-9: Patient Health Questionnaire-9.

### Intervention Effects

#### Primary Outcome

**Figure 2:**
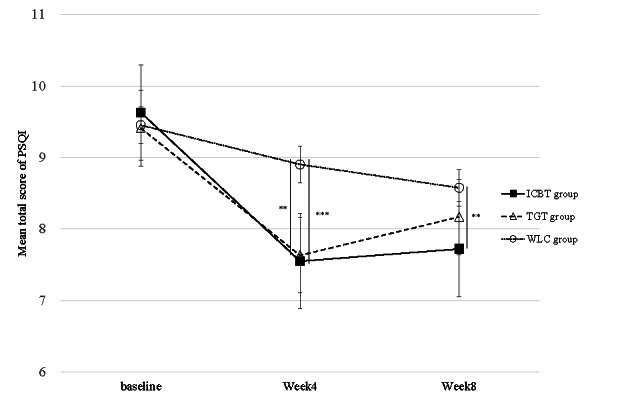
Means and SDs (raw data) for the primary outcome and improvement in the Pittsburgh Sleep Quality Index (PSQI) score. ICBT: internet-based cognitive behavioral therapy; TGT: Three Good Things; WLC: waiting list control.

Figure 2 and Table 3 show the changes in the primary outcome score (PSQI) in each group, and Table 4 shows the comparison of the changes in the primary outcome PSQI between the intervention and control groups. At week 4, the adjusted mean change from baseline in the ICBT group compared with the WLC group was −1.56 (95% CI −2.52 to −0.59; *P*<.001), and the adjusted mean change from baseline in the TGT group compared with the WLC group was −1.15 (95% CI −2.08 to −0.23; *P*=.002), indicating that both the ICBT and TGT groups had a significant reduction in their PSQI scores compared with the WLC group.

**Table 3:** Changes in primary outcome (PSQI) in the 3 groups.

Assigned group (week 4)	Estimation (SE; 95% CI)	*P* value
ICBT^a^	−2.19 (0.29; −2.76 to −1.63)	<.001
TGT^b^	−1.79 (0.26; −2.31 to –1.27)	<.001
WLC^c^	−0.64 (0.25; −1.14 to −0.14)	.01

^a^ICBT: internet-based cognitive behavioral therapy.

^b^TGT: Three Good Things.

^c^WLC: waiting list control.

**Table 4:** Changes in the secondary outcome among the 3 groups.

Secondary outcomes and assigned group				Estimation (SE)		*P* value		95% CI
**PSQI^a^**								
	**Week 8**							
		ICBT^b^	−2.09 (0.29)		<.001		−2.66 to −1.51	
		TGT^c^	−1.34 (0.26)		<.001		−1.86 to −0.82	
		WLC^d^	−0.99 (0.25)		<.001		−1.49 to −0.50	
**SOL^e^**								
	**Week 4**							
		ICBT	−19.11 (2.55)		<.001		−24.13 to −14.08	
		TGT	−13.26 (2.38)		<.001		−17.95 to −8.56	
		WLC	−7.38 (2.29)		.001		−11.88 to −2.87	
	**Week 8**							
		ICBT	−15.13 (2.64)		<.001		−20.33 to −9.94	
		TGT	−12.39 (2.40)		<.001		−17.11 to −7.67	
		WLC	−7.40 (2.26)		.001		−11.86 to −2.94	
**TST^f^**								
	**Week 4**							
		ICBT	37.40 (5.27)		<.001		27.01 to 47.78	
		TGT	25.02 (4.89)		<.001		15.39 to 34.66	
		WLC	4.32 (4.72)		.36		−4.98 to 13.63	
	**Week 8**							
		ICBT	31.73 (5.43)		<.001		21.03 to 42.43	
		TGT	14.25 (4.91)		.004		4.57 to 23.93	
		WLC	14.23 (4.68)		.003		5.00 to 23.45	
**Sleep efficiency**								
	**Week 4**							
		ICBT	5.85 (1.37)		<.001		3.15 to 8.55	
		TGT	6.37 (1.28)		<.001		3.86 to 8.89	
		WLC	2.90 (1.22)		.02		0.50 to 5.31	
	**Week 8**							
		ICBT	6.73 (1.42)		<.001		3.93 to 9.52	
		TGT	3.77 (1.30)		.004		1.19 to 6.34	
		WLC	2.19 (1.21)		.07		−0.19 to 4.57	
**AIS^g^**								
	**Week 4**							
		ICBT	−2.11 (0.37)		<.001		−2.83 to −1.39	
		TGT	−2.75 (0.34)		<.001		−3.43 to −2.08	
		WLC	−0.49 (0.32)		<.001		−1.12 to 0.15	
	**Week 8**							
		ICBT	−1.82 (0.38)		<.001		−2.57 to −1.08	
		TGT	−1.62 (0.34)		<.001		−2.29 to −0.95	
		WLC	−1.21 (0.32)		<.001		−1.84 to −0.57	
**GAD-7^h^**								
	**Week 4**							
		ICBT	0.17 (0.30)		.58		−0.43 to 0.77	
		TGT	−0.44 (0.28)		.12		−1.00 to 0.12	
		WLC	0.39 (0.27)		.15		−0.14 to 0.91	
	**Week 8**							
		ICBT	0.07 (0.31)		.83		−0.55 to 0.69	
		TGT	0.11 (0.28)		.70		−0.45 to 0.67	
		WLC	0.07 (0.27)		.79		−0.45 to 0.60	
**PHQ-9^i^**								
	**Week 4**							
		ICBT	−0.32 (0.35)		.37		−1.02 to 0.38	
		TGT	−0.40 (0.33)		.23		−1.05 to 0.25	
		WLC	1.38 (0.31)		<.001		0.77 to 2.00	
	**Week 8**							
		ICBT	−0.27 (0.37)		.46		−0.99 to 0.45	
		TGT	−0.03 (0.33)		.93		−0.69 to 0.63	
		WLC	0.38 (0.31)		.22		−0.24 to 1.00	
**CES-D^j^**								
	**Week 4**							
		ICBT	0.40 (0.36)		.27		−0.30 to 1.10	
		TGT	0.54 (0.33)		.10		−0.11 to 1.20	
		WLC	0.53 (0.31)		.09		−0.09 to 1.14	
	**Week 8**							
		ICBT	0.47 (0.37)		.20		−0.25 to 1.19	
		TGT	0.52 (0.33)		.12		−0.13 to 1.18	
		WLC	−0.51 (0.31)		.11		−1.12 to 0.11	

^a^PSQI: Pittsburgh Sleep Quality Index.

^b^ICBT: internet-based cognitive behavioral therapy.

^c^TGT: Three Good Things.

^d^WLC: waiting list control.

^e^SOL: sleep onset latency.

^f^TST: total sleep time.

^g^AIS: Athens Insomnia Scale.

^h^GAD-7: Generalized Anxiety Disorder-7.

^i^PHQ-9: Patient Health Questionnaire-9.

^j^CES-D: Center for Epidemiologic Studies–Depression Scale.

#### Secondary Outcomes

Table 4 shows the changes in the secondary evaluation items for each group. Table 5 shows a comparison of the changes in secondary outcomes between the intervention and control groups.

**Table 5:** Comparison of changes in the secondary outcome between intervention groups and control group.

Secondary outcomes and assigned group			Control group	Estimation (SE)	Adjusted *P* value^a^	95% CI (adjusted)
**PSQI^b^**						
	**Week 8**					
		ICBT^c^	WLC^d^	−1.09 (0.38)	.02	−2.07 to −0.12
		TGT^e^	WLC	−0.35 (0.36)	.81	−1.27 to 0.57
**SOL^f^**						
	**Week 4**					
		ICBT	WLC	−11.73 (3.40)	.003	−20.36 to −3.10
		TGT	WLC	−5.88 (3.30)	.27	−14.24 to 2.48
	**Week 8**					
		ICBT	WLC	−7.74(3.45)	.10	−16.48 to 1.01
		TGT	WLC	−5.00 (3.29)	.42	−13.34 to 3.34
**TST^g^**						
	**Week 4**					
		ICBT	WLC	33.07 (7.07)	<.001	15.17 to 50.98
		TGT	WLC	20.70 (6.79)	.01	3.51 to 37.89
	**Week 8**					
		ICBT	WLC	17.50 (7.15)	.06	−0.63 to 35.63
		TGT	WLC	0.02 (6.77)	.99	−17.13 to 17.17
**Sleep efficiency**						
	**Week 4**					
		ICBT	WLC	2.94 (1.83)	.36	−1.70 to 7.59
		TGT	WLC	3.47 (1.76)	.18	−1.00 to 7.94
	**Week 8**					
		ICBT	WLC	4.53 (1.85)	.06	−0.18 to 9.25
		TGT	WLC	1.57 (1.77)	.85	−2.92 to 6.07
**AIS^h^**						
	**Week 4**					
		ICBT	WLC	−1.62 (0.48)	.004	−2.85 to −0.39
		TGT	WLC	−2.27 (0.47)	<.001	−3.46 to −1.08
	**Week 8**					
		ICBT	WLC	−0.62 (0.49)	.61	−1.87 to 0.63
		TGT	WLC	−0.41 (0.47)	.85	−1.60 to 0.78
**GAD-7^i^**						
	**Week 4**					
		ICBT	WLC	−0.22 (0.40)	.98	−1.24 to 0.80
		TGT	WLC	−0.82 (0.39)	.14	−1.81 to 0.16
	**Week 8**					
		ICBT	WLC	−0.01 (0.41)	.99	−1.05 to 1.03
		TGT	WLC	0.04 (0.39)	.99	−0.95 to 1.03
**PHQ-9^j^**						
	**Week 4**					
		ICBT	WLC	−1.70 (0.47)	.001	−2.90 to −0.51
		TGT	WLC	−1.78 (0.45)	<.001	−2.94 to −0.63
	**Week 8**					
		ICBT	WLC	−0.65 (0.48)	.53	−1.86 to 0.56
		TGT	WLC	−0.41 (0.46)	.84	−1.57 to 0.74
**CES-D^k^**						
	**Week 4**					
		ICBT	WLC	−0.13 (0.47)	.99	−1.32 to 1.06
		TGT	WLC	0.02 (0.45)	.99	−1.13 to 1.17
	**Week 8**					
		ICBT	WLC	0.98 (0.48)	.16	−0.23 to 2.19
		TGT	WLC	1.03 (0.45)	.10	−0.12 to 2.18

^a^Dunnett–Hsu.

^b^PSQI: Pittsburgh Sleep Quality Index.

^c^ICBT: internet-based cognitive behavioral therapy.

^d^WLC: waiting list control.

^e^TGT: Three Good Things.

^f^SOL: sleep onset latency.

^g^TST: total sleep time.

^h^AIS: Athens Insomnia Scale.

^i^GAD-7: Generalized Anxiety Disorder-7.

^j^PHQ-9: Patient Health Questionnaire-9.

^k^CES-D: Center for Epidemiologic Studies–Depression Scale.

At 4 weeks, the adjusted mean change from baseline in the ICBT group compared with the WLC group was −11.73 for SOL (95% CI −20.36 to −3.10; *P*=.003), 17.50 for TST (95% CI 15.17-50.98; *P*<.001), −1.62 for AIS (95% CI −2.85 to −0.39; *P*=.004), and −1.70 for PHQ-9 (95% CI −2.90 to −0.51; *P*=.002). There was a significant improvement in the ICBT group compared with the WLC group in SOL, TST, AIS, and PHQ-9 scores at 4 weeks.

At 4 weeks, the adjusted mean change from baseline in the TGT group compared with the WLC group was 20.70 for TST (95% CI 3.51-37.89; *P*=.01), −2.27 for AIS (95% CI −3.46 to −1.08; *P*<.001), and −1.78 for PHQ-9 (95% CI −2.94 to −0.63; *P*=.001). A significant improvement in the TGT group compared with the WLC group in TST, AIS, and PHQ-9 scores was observed at 4 weeks.

At 8 weeks, the adjusted mean change from baseline in the ICBT group compared with the WLC group was −1.09 for PSQI (95% CI −2.07 to −0.12; *P*=.02). There was a significant improvement in the ICBT group compared with the WLC group in PSQI at 8 weeks.

At 8 weeks, the adjusted mean change from baseline in the TGT group compared with the WLC group did not show a significant difference.

#### Effectiveness of the Intervention

Table 6 shows the standardized change from baseline to the postintervention survey (at 4 and 8 weeks). Immediately after the intervention, at 4 weeks, the primary outcome effect size (Hedge g) for the PSQI was 0.81 in the ICBT group (95% CI 6.90-8.20) and 0.76 in the TGT group (95% CI 7.06-8.20).

**Table 6:** Standardized changes of baseline to postintervention outcomes.

Outcomes		Values, mean (SD)				Effect size (95% CI)^a,b^		
		ICBT^c^	TGT^d^	WLC^e^	ICBT		TGT	WLC
**PSQI^f^**								
	Baseline	9.63 (2.32)	9.41 (2.05)	9.45 (2.20)	9.10-10.15		8.97-9.84	9.02-9.88
	Week 4	7.55 (2.79)	7.63 (2.62)	8.90 (2.89)	0.81 (6.90-8.20)		0.76 (7.06-8.20)	0.22 (8.30-9.50)
	Week 8	7.72 (3.13)	8.17 (2.66)	8.57 (2.93)	0.70 (6.96-8.48)		0.52 (7.59-8.75)	0.34 (7.97-9.18)
**SOL^g^**								
	Baseline	41.75 (27.88)	47.78 (40.64)	41.23 (34.96)	35.50-47.99		39.17-56.39	34.40-48.07
	Week 4	23.41 (22.55)	32.61 (27.20)	34.87 (37.29)	0.72 (18.18-28.63)		0.44 (26.70-38.51)	0.18 (27.10-42.63)
	Week 8	27.90 (26.44)	33.72 (29.90)	34.43 (32.74)	0.51 (21.50-34.30)		0.39 (27.19-40.25)	0.20 (27.72-41.13)
**TST^h^**								
	Baseline	318.56 (63.66)	323.81 (52.11)	319.93 (61.73)	304.30-332.82		312.77-334.85	307.87-332.00
	Week 4	360.84 (60.28)	350.18 (61.20)	323.57 (65.53)	0.68 (346.87-374.80)		0.46 (336.90-363.46)	0.06 (309.92-337.22)
	Week 8	355.90 (62.76)	338.67 (58.26)	333.27 (64.46)	0.59 (340.71-371.09)		0.27 (325.95-351.40)	0.21 (320.06-346.47)
**Sleep efficiency**								
	Baseline	82.12 (15.24)	85.36 (10.93)	84.25 (14.68)	78.64-85.61		83.02-87.70	81.34-87.17
	Week 4	89.07 (11.77)	90.68 (8.96)	86.76 (13.41)	0.51 (86.26-91.87)		0.53 (88.73-92.64)	0.18 (83.92-89.60)
	Week 8	89.79 (9.04)	87.62 (10.86)	86.67 (14.75)	0.60 (87.52-92.07)		0.21 (85.15-90.08)	0.16 (83.61-89.72)
**AIS^i^**								
	Baseline	10.19 (2.95)	9.95 (2.66)	10.24 (2.90)	9.53-10.85		9.39-10.52	9.68-10.81
	Week 4	8.14 (3.18)	7.29 (3.67)	9.72 (3.67)	0.67 (7.40-8.87)		0.84 (6.49-8.08)	0.16 (8.97-10.47)
	Week 8	8.47 (3.89)	8.47 (3.59)	9.09 (3.28)	0.50 (7.53-9.41)		0.47 (7.69-9.25)	0.37 (8.43-9.76)
**GAD-7^j^**								
	Baseline	2.67 (2.37)	2.91 (2.41)	2.66 (2.37)	2.14-3.20		2.40-3.42	2.20-3.12
	Week 4	2.92 (3.29)	2.45 (2.49)	3.02 (3.18)	0.09 (2.16-3.68)		0.19 (1.91-2.99)	0.13 (2.37-3.67)
	Week 8	2.84 (3.20)	3.00 (3.03)	2.80 (2.93)	0.06 (2.06-3.61)		0.03 (2.34-3.66)	0.05 (2.20-3.40)
**PHQ-9^k^**								
	Baseline	3.99 (2.23)	4.18 (2.38)	4.08 (2.26)	3.49-4.49		3.68-4.69	3.64-4.52
	Week 4	3.68 (2.88)	3.83 (2.94)	5.33 (4.46)	0.12 (3.01 to 4.34)		0.13 (3.20-4.47)	0.36 (4.42-6.24)
	Week 8	3.69 (3.29)	4.28 (3.18)	4.29 (3.34)	0.11 (2.90 to 4.49)		0.03 (3.58-4.97)	0.08 (3.59-5.00)
**CES-D^l^**								
	Baseline	7.01 (3.46)	7.10 (3.38)	7.02 (3.74)	6.24-7.79		6.39-7.82	6.29-7.75
	Week 4	7.32 (3.81)	7.60 (3.64)	7.55 (3.65)	0.09 (6.44-8.21)		0.14 (6.81-8.38)	0.14 (6.80-8.30)
	Week 8	7.41 (3.33)	7.54 (3.67)	6.51 (4.09)	0.12 (6.60-8.22)		0.12 (6.74-8.34)	0.13 (5.67-7.34)

^a^Hedges *g* (effect size; 95% CI lower limit to upper limit of each outcome).

^b^Effect size values for baseline were not applicable; hence, only 95% CI values have been reported.

^c^ICBT: internet-based cognitive behavioral therapy.

^d^TGT: Three Good Things.

^e^WLC: waiting list control.

^f^PSQI: Pittsburgh Sleep Quality Index.

^g^SOL: sleep onset latency.

^h^TST: total sleep time.

^i^AIS: Athens Insomnia Scale.

^j^GAD-7: Generalized Anxiety Disorder-7.

^k^PHQ-9: Patient Health Questionnaire-9.

^l^CES-D: Center for Epidemiologic Studies–Depression Scale.

The secondary outcome effect sizes (Hedge g) were as follows: 0.72 for SOL in the ICBT group (95% CI 18.18-28.63), 0.68 for TST in the ICBT group (95% CI of 346.87-374.80), 0.51 for SE in the ICBT group (95% CI 86.26-91.87) and 0.53 in the TGT group (95% CI 88.73-92.64), and 0.67 for AIS in the ICBT group (95% CI 7.40-8.87) and 0.84 in the TGT group (95% CI 6.49-8.08).

After the follow-up period, at 8 weeks, the secondary outcome effect sizes (Hedges *g*) were as follows: 0.70 for PSQI in the ICBT group (95% CI 6.96-8.48) and 0.52 in the TGT group (95% CI 7.59-8.75), 0.51 for SOL in the ICBT group (95% CI 21.50-34.30), 0.59 for TST in the ICBT group (95% CI 340.71-371.09), 0.60 for SE in the ICBT group (95% CI 87.52-92.07), and 0.50 for AIS in the ICBT group (95% CI 7.53-9.41).

## Discussion

### Principal Findings

The results of this study suggest that a self-help internet intervention without email support is effective as a noninvasive self-medication for adults with insomnia. The ICBT group showed a significant change of −6.11 in the PSQI score. The participant characteristics of this study differed significantly from those of similar previous studies. A previous study by van Straten et al [22] showed the efficacy of guided ICBT using an RCT design, in which 1500 patients with insomnia were invited by email, and 118 patients were assigned to the ICBT group (n=59) and a WLC group (n=59). However, the reported baseline mean PSQI score in the ICBT group in that study was relatively severe (12.4, SD 2.1). The mean change in PSQI scores in the ICBT group was −3.5, and in the WLC group it was −0.1. Our previous study of ICBT guided by email support reported a severe baseline PSQI mean score of 13.5 (SD 2.7); furthermore, participants had persistent symptoms despite taking sleep medications such as benzodiazepines [9].

In this study, the unguided ICBT group showed a change of −2.19, the TGT group showed a change of −1.79, and the WLC group showed a change of −0.64; the mean change in PSQI scores was smaller than those reported by van Straten et al [22] and Sato et al [9]. This could be because of the baseline average score of PSQI being 9.8, which indicates relatively mild insomnia. In addition, given that the ICBT was unguided, participant motivation to continue in this study may not have been well-supported.

The TGT group showed a significant decrease in PSQI scores compared with the WLC group only at the end of the program, that is, at the 4-week time point. To the best of our knowledge, no previous studies have examined the effects of TGT on insomnia through an RCT. However, as no significant improvement in positive emotions (indicated by the actual items on the CES-D) was observed in the TGT group, this casts doubt on the existence of a mechanism for the improvement of insomnia through the improvement of positive emotions. Contrary to previous studies, the TGT exercise did not lead to the improvement of positive emotions, and thus, it is unclear whether TGT really contributes to the improvement of happiness.

In this study, comparing the ICBT group and the TGT group was not the original purpose; however, the number of participants who received >75% of the intervention in the ICBT group was 70% (55/79) of participants compared with 90% (79/88) of participants who received >75% of the intervention in the TGT group. This could have been because of difficulties with regular participation in the ICBT program.

At the 4- and 8-week follow-ups, the ICBT group showed a significant improvement in PSQI scores over the WLC group, whereas the TGT group did not show a significant improvement over the WLC group. Therefore, ICBT could have longer-term effectiveness. If further research shows that ICBT results in sustained effects on insomnia, and TGT is easier to participate in more regularly, it may be necessary to consider an unguided intervention that combines both ICBT and TGT to achieve optimal outcomes.

### Limitations

In this study, there were errors in the calculation set by the internet research company that conducted the web questionnaire for PSQI, PHQ-9, and GAD-7 scores, which may have affected the eligible participants. In future studies, the distribution of inclusion and exclusion criteria should be carefully monitored to avoid similar mistakes.

Some participants in the ICBT and TGT groups did not participate more than once. However, in the WLC group, all participants were included in the final analysis. To ensure consistency with the ICBT and TGT groups in future research, it will be necessary to select those who have participated at least once in the WLC group for inclusion in the final analysis, or alternatively a placebo sham program could be implemented.

### Conclusions

In conclusion, this RCT provided evidence that 4 weeks of unguided ICBT and TGT exercise for adults with insomnia, both administered as self-help internet interventions without email support, may improve insomnia symptoms compared with the WLC group. The findings also suggest that the effects of ICBT may last longer than TGT, whereas TGT may be easier to participate in more regularly. However, this study experienced errors during the selection process. Further research is warranted to examine the effectiveness of a combined intervention of ICBT and TGT for insomnia.

## Appendix

Multimedia Appendix 1 CONSORT-eHEALTH checklist (V 1.6.1).

## Abbreviations

AIS: Athens Insomnia Scale
CBT: cognitive behavioral therapy
CES-D: Center for Epidemiologic Studies–Depression Scale
CONSORT: Consolidated Standards of Reporting Trials
CONSORT-EHEALTH: Consolidated Standards of Reporting Trials of Electronic and Mobile Health Applications and Online Telehealth
FAS: full analysis set
GAD-7: Generalized Anxiety Disorder-7
ICBT: internet-based cognitive behavioral therapy
PHQ-9: Patient Health Questionnaire-9
PPS: per-protocol set
PSQI: Pittsburgh Sleep Quality Index
RCT: randomized controlled trial
RIETI: Research Institute of Economy, Trade, and Industry
SE: sleep efficiency
SOL: sleep onset latency
TGT: Three Good Things
TST: total sleep time
UC: usual care
WLC: waiting list control

## References

[1] (2013). Diagnostic and Statistical Manual of Mental Disorders (DSM-5®).

[2] Ohayon MM (2002). Epidemiology of insomnia: what we know and what we still need to learn. Sleep Med Rev.

[3] Komada Y, Nomura T, Kusumi M, Nakashima K, Okajima I, Sasai T, Inoue Y (2011). Correlations among insomnia symptoms, sleep medication use and depressive symptoms. Psychiatry Clin Neurosci.

[4] Kim K, Uchiyama M, Okawa M, Liu X, Ogihara R (2000). An epidemiological study of insomnia among the Japanese general population. Sleep.

[5] Hafner M, Stepanek M, Taylor J, Troxel WM, van Stolk C (2017). Why sleep matters-the economic costs of insufficient sleep: a cross-country comparative analysis. Rand Health Q.

[6] Yamauchi M (2006). [Diagnosis of and therapy for sleep disorders in primary care]. Seishin Shinkeigaku Zasshi.

[7] Seligman ME, Steen TA, Park N, Peterson C (2005). Positive psychology progress: empirical validation of interventions. Am Psychol.

[8] Sato D, Yoshinaga N, Nagai E, Hanaoka H, Sato Y, Shimizu E (2018). Randomised controlled trial on the effect of internet-delivered computerised cognitive-behavioural therapy on patients with insomnia who remain symptomatic following hypnotics: a study protocol. BMJ Open.

[9] Sato D, Yoshinaga N, Nagai E, Nagai K, Shimizu E (2019). Effectiveness of internet-delivered computerized cognitive behavioral therapy for patients with insomnia who remain symptomatic following pharmacotherapy: randomized controlled exploratory trial. J Med Internet Res.

[10] Ong AD, Kim S, Young S, Steptoe A (2017). Positive affect and sleep: a systematic review. Sleep Med Rev.

[11] Seligman M (2011). Flourish: a new understanding of happiness and wellbeing: the practical guide to using positive psychology to make you happier and healthier.

[12] Buysse DJ, Reynolds CF, Monk TH, Berman SR, Kupfer DJ (1989). The Pittsburgh Sleep Quality Index: a new instrument for psychiatric practice and research. Psychiatry Res.

[13] Doi Y, Minowa M, Uchiyama M, Okawa M, Kim K, Shibui K, Kamei Y (2000). Psychometric assessment of subjective sleep quality using the Japanese version of the Pittsburgh Sleep Quality Index (PSQI-J) in psychiatric disordered and control subjects. Psychiatry Res.

[14] Soldatos CR, Dikeos DG, Paparrigopoulos TJ (2000). Athens Insomnia Scale: validation of an instrument based on ICD-10 criteria. J Psychosom Res.

[15] Okajima I, Nakajima S, Kobayashi M, Inoue Y (2013). Development and validation of the Japanese version of the Athens Insomnia Scale. Psychiatry Clin Neurosci.

[16] Spitzer RL, Kroenke K, Williams JB, Löwe B (2006). A brief measure for assessing generalized anxiety disorder: the GAD-7. Arch Intern Med.

[17] Doi Satomi, Ito Masaya, Takebayashi Yoshitake, Muramatsu Kumiko, Horikoshi Masaru (2018). Factorial Validity and Invariance of the 7-Item Generalized Anxiety Disorder Scale (GAD-7) Among Populations With and Without Self-Reported Psychiatric Diagnostic Status. Front Psychol.

[18] Kroenke K, Spitzer RL, Williams JB (2001). The PHQ-9: validity of a brief depression severity measure. J Gen Intern Med.

[19] Muramatsu K, Miyaoka H, Kamijima K, Muramatsu Y, Yoshida M, Otsubo T, Gejyo F (2007). The patient health questionnaire, Japanese version: validity according to the mini-international neuropsychiatric interview-plus. Psychol Rep.

[20] Radloff LS (1977). The CES-D Scale: a self-report depression scale for research in the general population. Applied Psychol Measurement.

[21] Shima S, Shikano T, Kitamura T, Asai M (1985). New self-rating scale for depression. Clin Psychiatry.

[22] van Straten A, Emmelkamp J, de Wit J, Lancee J, Andersson G, van Someren EJ, Cuijpers P (2014). Guided internet-delivered cognitive behavioural treatment for insomnia: a randomized trial. Psychol Med.

